# Supplementary material for: Content-rich biological network constructed by mining PubMed abstracts
Source: BMC Bioinformatics. 2004 Oct 8;5:147. doi: 10.1186/1471-2105-5-147 (PMC528731; doi:10.1186/1471-2105-5-147)
Supplement: Additional File 2 — The original results of the above study (non-essential files are deleted to keep the file size under the limit set by BMC bioinformatics). [file 1471-2105-5-147-S2.bz2 › chilibotAdditionalFile2/dip05/768ID10633096E18159/html/FECA_FECR.html]

 


 **FECA** and **FECR** 
  
Found 16 abstracts in PubMed, retrieved 05.  
 

 What does Google say? 
 PDF only 
| .edu only 

---

**Interactive relationship** (e.g. stimulation, inhibition, etc)

**Neutral relationship**- Sites of Interaction between the  **FecA**  and  **FecR**  Signal Transduction Proteins of Ferric Citrate Transport in Escherichia coli K 12.  Ref: 12813067 J Bacteriol, 2003
- In the same C terminal region, amino acid residues important for the interaction of  **FecR**  with  **FecA**  were identified by random and site directed mutagenesis.  Ref: 12813067 J Bacteriol, 2003
- The amino proximal end of  **FecA**  interacts with the carboxy proximal end of  **FecR**  in the periplasm.  Ref: 12732308 Curr Opin Microbiol, 2003
- **FecR**  receives the signal in the periplasm from the outer membrane protein  **FecA**  loaded with ferric citrate, transmits the signal across the cytoplasmic membrane, and converts FecI in the cytoplasm to an active sigma factor.  Ref: 12813067 J Bacteriol, 2003
- The data showed that  **FecR**  interacts in the periplasm with  **FecA**  to confer ferric citrate induced transcription of the fec transport genes and identified sites in  **FecR**  and  **FecA**  that are important for signal transduction.  Ref: 12813067 J Bacteriol, 2003
- Three randomly generated inactive  **FecR**  mutants,  **FecR**  L254E,  **FecR**  L269G, and  **FecR**  F284L, were suppressed to different degrees by the mutants  **FecA**  G39R and  **FecR**  D43E.  Ref: 12813067 J Bacteriol, 2003

**Non-interactive relationship** (e.g. studied together, co-existance, homology, etc.)

- One  **FecR**  mutant,  **FecR**  D138E, V197A, induced  **fecA**  promoter directed transcription constitutively in the absence of ferric citrate and bound more strongly than wild type  **FecR**  to  **FecA** .  Ref: 12813067 J Bacteriol, 2003
- Bound ferric citrate does not have to be transported but initiates a signal that is transmitted by  **FecA**  across the outer membrane and by  **FecR**  across the cytoplasmic membrane into the cytoplasm, where the FecI extracytoplasmic function ECF sigma factor becomes active.  Ref: 11114913 J Bacteriol, 2001
- The degree of residual binding of  **FecR**  mutant proteins to  **FecA**  was correlated with the degree of transcription initiation in response to ferric citrate in the culture medium.  Ref: 12813067 J Bacteriol, 2003
- In this study, it was shown through the use of a bacterial two hybrid system that, in the periplasm, the C terminal  **FecR**  237 317 fragment interacts with the N terminal  **FecA**  1 79 fragment.  Ref: 12813067 J Bacteriol, 2003
- By analogy with the FecI  **FecR**   **FecA**  and PupI PupR PupB systems, our data suggest that bfrZ expression is inducible by binding of the cognate ligand to BfrZ and transduction of a signal through the envelope.  Ref: 11292812 J Bacteriol, 2001
- Three specific proteins are involved  **FecA**  in the outer membrane,  **FecR**  in the cytoplasmic membrane, and FecI in the cytoplasm.  Ref: 12732308 Curr Opin Microbiol, 2003
